# Effect of preconditioning and postoperative hyperbaric oxygen therapy on colonic anastomosis healing with and without ischemia in rats ^1^

**DOI:** 10.1590/s0102-865020200050000003

**Published:** 2020-06-22

**Authors:** José Luiz Fontoura-Andrade, Leonardo Mendes Pinto, Fabiana Pirani Carneiro, João Batista de Sousa

**Affiliations:** I Fellow Master degree, Postgraduate Program in Medical Sciences, School of Medicine, Universidade de Brasília (UnB). DVM Laboratory Animal Research Unit, Hospital das Forças Armadas (HFA), Brasilia-DF, Brazil. Conception and design of the study, technical procedures, acquisition and interpretation of data, statistics analysis, manuscript preparation and writing, critical revision, final approval.; Laboratory Animal Research Unit, Hospital das Forças Armadas, Brasilia, DF, Brazil; II Head, Hyperbaric Medicine Section, HFA, Brasilia-DF, Brazil. Conception and design of the study, technical procedures, critical revision, final approval.; III PhD, Associate Professor, Division of Pathology, School of Medicine, UnB, Brasilia-DF, Brazil. Macroscopic and histopathological examinations, interpretation of data, critical revision, final approval.; IV PhD, Associate Professor, Division of Surgery, School of Medicine, UnB, Brasilia-DF, Brazil. Conception and design of the study; acquisition, analysis and interpretation of data; manuscript preparation and writing; critical revision, final approval.

**Keywords:** Anastomotic Leak, Hyperbaric Oxygenation, Colon, Rats

## Abstract

**Purpose:**

To investigate the effect of hyperbaric oxygen therapy on colonic anastomosis healing with and without ischemia in rats.

**Methods:**

Forty female rats underwent segmental resection of 1 cm of the left colon followed by end-to-end anastomosis. They were randomly assigned to four groups (n=10 each), a sham group; two groups were submitted to Hyperbaric Oxygen therapy (HBOT) with and without induced ischemia and the induced ischemia group without HBOT. The HBOT protocol evaluated was 100% O2 at 2.4 Atmosphere absolute pressure (ATA) for 60 minutes, two sessions before as a preconditioning protocol and three sessions after the operation. Clinical course and mortality were monitored during all experiment and on the day of euthanasia on the fourth day after laparotomy. Macroscopic appearance of the abdominal cavity were assessed and samples for breaking strength of the anastomosis and histopathological parameters were collected.

**Results:**

There was no statistically significant difference in mortality or anastomosis leak between the four experimental groups. Anastomosis breaking strength was similar across groups.

**Conclusion:**

The HBOT protocol tested herein at 2.4 ATA did not affect histopathological and biomechanical parameters of colonic anastomotic healing, neither the clinical outcomes death and anastomosis leak on the fourth day after laparotomy.

## Introduction

Complications of colorectal anastomosis have a relevant incidence and are often severe. In addition to their contribution to an undesirably high mortality rate, further implications include prolonged hospital stay, substantially increased treatment cost, and perpetuation of functional sequelae. The most fearsome complications are infection and anastomotic dehiscence^1^ . Several drugs have been investigated in the search to reduce the incidence of these complications. In 2014, Oines *et al* .^2^ published a meta-analysis reporting experiments with 56 therapeutic agents, of which only seven met their inclusion criteria: iloprost, tacrolimus, erythropoietin, growth hormone, broad-spectrum matrix metalloproteinase inhibitors, insulin-like growth factor-1, and hyperbaric oxygen therapy (HBOT). These therapeutic agents experimentally increased the pressure required for disruption of colon anastomosis.

Most of the published data refer to postoperative HBOT sessions; few authors reported results with preoperative or combined pre and postoperative HBOT^3^ .

HBOT after the procedure or when the lesion is already in place is a well-established tool in clinical practice; the most commonly studied approach to date is postoperative HBOT^4 - 8^ . Benefits of HBOT preceding the intervention have also been reported, in surgery^9^ , in radiotherapy^10 , 11^ , and in procedures related to experimentally induced neurological lesions in animal models^5 , 12^ .

Preconditioning is an intervention that can potentially lessen sequelae of a subsequent ischemia insult. We have assumed that previous HBOT sessions could provide preconditioning and could offer some protection to experimental ischemic colon resection.

Within this context, the present study was designed to assess the effect of both preconditioning and postoperative HBOT sessions on colonic healing, with and without induced ischemia, after experimental ischemic colon resection and anastomosis in rats.

## Methods

This study was approved by the Hospital das Forças Armadas Animal Care and Use Committee under the registration number 60550.008623/2016-00. The “Animal Research: Reporting in vivo experiments” (ARRIVE) guidelines were followed in designing, conducting, and reporting the experiment^13 , 14^ . All procedures with animals were carried out in accordance with the UK Animals (Scientific Procedures) Act, 1986, and associated guidelines and EU Directive 2010/63/EU and International Guiding principles for biomedical research involving animals by Council for International Organization of Medical Sciences-CIOMS.

Forty female LEW/HsdUnib rats were acquired from the Specific Pathogen-Free Animal Production Division of the Multidisciplinary Center for Biological Research in Laboratory Animal Science, Campinas State University, Campinas, Brazil. The health monitoring program of this supplier is executed in accordance with the international recommendations of the Federation of Laboratory Animal Science Associations (FELASA). All 40 rats were used for experiments. Females were chosen since there is scarce published data, male animal model far outnumber experiments on female animal subjects.

The mean (standard deviation -SD) weight on the day of surgery was 218.28 (12.20) g, and the median (first quartile-third quartile) age was 128 (125-131) days. Rats were distributed in blocks of four animals; one animal from each block was randomly assigned to each of the experimental groups, in standardized cages. Autoclaved wood shavings were used for bedding. The environmental conditions were: mean (SD) ambient temperature 25.3°C (0.68), mean (SD) humidity 48% (8.73), 12-hour light/dark cycles (inverted with natural light, with the lights turned automatically off at 8:00 AM and on at 8:00 PM daily). All animals had access to drinking water *ad libitum* and were fed commercial chow for laboratory rats (Presence Ratos e Camundongos™, Alimentos de Paulínia, SP).

### Experimental groups

The following procedures were done in all groups: resection of a 1-cm segment of the left colon, end-to-end anastomosis, and reoperation on the fourth day after laparotomy. In addition to these procedures, the subjects were divided into groups as follows:

HBOT/Ischemia (n = 10): hyperbaric therapy + induced ischemia;

HBOT (n = 10): hyperbaric therapy + sham ischemia;

Ischemia (n = 10): induced ischemia + sham HBOT;

Control (n = 10): sham ischemia + sham HBOT.

### Hyperbaric oxygen therapy

HBOT was delivered in a Monoplace hyperbaric chamber (Model 2800, Sechrist Industries, Inc.). The experimental block was formed by one animal from each experimental group housed in a separate cage. Each block of four animals was assigned to the procedures under the same conditions. For HBOT sessions, each block was separated into two perforated plastic boxes (230 mm high, 210 mm wide, and 300 mm long with multiple perforations). The boxes containing animals from the HBOT and HBOT/Ischemia groups were exposed to HBOT inside the hyperbaric chamber, while boxes containing animals from the Control and Ischemia groups were left outside the hyperbaric chamber (sham HBOT). Six daily sessions were administered on afternoons. The sessions applied as preconditioning were on the first and second day of experiment. The third session was after the procedure. Three more sessions were administered on days fourth, fifth and sixth. All sessions consisted of hyperbaric hyperoxia with 100% oxygen at a maximal pressure of 2.4 atmospheres absolute (ATA) for 60 minutes. Both compression and decompression were conducted at a mean (SD) velocity of 0.07 (0.02) ATA/min. The chamber temperature was maintained between 22 and 25°C. Ambient light was dimmed for the comfort of the rats. All sessions began at the same time of day, between 2:00 PM and 3:00 PM, in order to minimize effects on the circadian cycle.

### Anesthesia and surgical procedures

All surgical materials were autoclaved before use. All rats were operated on the same time of day (morning shift), by the same surgeon on the morning of the third day of the experiment. Blocks of four animals were operated each day, with one subject from each experimental group, and the sequence of procedures was randomized within the blocks by blindly picking papers with codes in an envelope. General anesthesia was induced with midazolam 5 mg/kg and ketamine 75 mg/kg, mixed in the same syringe and administered intraperitoneally.

Immediately after intraperitoneal injection of the induction agents, the rats were placed back in their original boxes, in the company of the other animals in their respective blocks. Once immobility was detected, lubricating ophthalmic gel was applied onto the eyes and 10 mL of a mixture of warm saline solution and 5% glucose was injected subcutaneously into the dorsal region. Oxygen was administered for 5 minutes via mask. After this time, sevoflurane was administered at a concentration of 0.25 to 0.5% for anesthetic maintenance via a Mapleson E nonrebreathing system. Midline laparotomy was performed through an incision approximately 4 cm long on the sagittal plane of the abdomen, and the distal left colon was exposed.

In the Ischemia and HBOT/Ischemia groups, ischemia was induced as follows: Peyer’s patch and the peritoneal reflection were identified^15^ as the landmark for the procedure. A colon segment 2 cm cranial to the landmark was isolated and the venous-arterial branches that drained or irrigated this left colonic segment were electrocoagulated. The mesenteric vessels of the vasa recta were electrocoagulated with bipolar hemostatic forceps set to 10 W. Then, 1 cm of the middle third of this devascularized portion was resected, as described by Adas *et al* .^16^ . The colon was then repaired by a single-plane end-to-end anastomosis with continuous 6-0 polypropylene sutures placed with a 1.3 cm cylindrical needle (Prolene^®^, ETHICON), encompassing all layers of the intestinal wall. The abdominal wall was sutured in two layers (first peritoneum, muscle, and aponeurosis of the sagittal abdominal plane, secured with 5-0 polypropylene simple running sutures; the second including subcutaneous tissues only). The skin incision was also sutured with 5-0 polypropylene. For recovery from anesthesia, the animals were kept for 30 minutes in the anesthetic induction box on a warming blanket at 38°C and with supplemental oxygen, as described by Durães *et al* .^17^ . They were then returned to their respective cages.

The end-to-end anastomosis was sutured using a surgical microscope. All other procedures, including celiotomy, localization and exposure of the colon, electrocauterization, cavity closure, and skin sutures, were performed without the aid of a microscope.

Reoperation was performed four days after the operation, on the seventh day of the experiment, to evaluate the abdominal cavity and the anastomosis site, collect abdominal scar tissue samples, and harvest the colon segment containing the anastomotic scar. Animals were anesthetized by intraperitoneal injection of tramadol 12.5 mg/kg, midazolam 5 mg/kg, and ketamine 75 mg/kg, mixed in the same syringe and applied at the same time. The abdominal cavity was opened by resection of a rectangular segment of the abdominal wall that contained the previous laparotomy scar. After extensive exposure of the cavity, a checklist was followed to search for signs of diffuse peritonitis, abscesses, blockage, dehiscence, or leakage of the anastomosis, signs of intestinal obstruction, and adhesions. The latter were quantified using the Nair score. Finally, the portion of the left colon containing the anastomosis was resected.

Immediately after resection, with the animal under anesthesia, euthanasia was performed by transection of the vena cava.

### Macroscopic evaluation on reoperation

After exposure of the abdominal cavity, signs of peritonitis, abscess, or anastomotic dehiscence were evaluated. The total amount of intra-abdominal adhesion was evaluated using the Nair score^24^:

0- no adhesion;
1-single band between viscera or between viscera and abdominal wall;
2- two bands between viscera or between viscera and abdominal wall;
3- more than two bands between viscera or between viscera and abdominal wall, or the entire intestine forming a mass adhering to the abdominal wall;
4- viscera directly attached to the abdominal wall, regardless of number or extent of bands.

The surgical specimen was opened through the antimesenteric border and divided into longitudinal segments for further analysis of the tensile strength and histopathological study. The samples intended for tensile strength were frozen in saline at -80ºC.

### Breaking strength measurement

To mechanically evaluate anastomotic healing, breaking strength in Newtons (N) was measured in a VersaTest universal testing machine (Mecmesin VersaTest, United Kingdom) with a 2500 N dynamometer (Mecmesin AFG 2500N, United Kingdom).

A colon segment without any adhesions was attached at the ends of the dynamometer clamp, with the suture line equidistant from the attachment points. At a constant crosshead speed of 25 mm/min, each claw was moved on opposite directions along the same axis until the suture line ruptured. The highest value was recorded by the device and logged as the breaking strength ( Table 1 ).

**Table 1:** Statistical results of breaking strength (in Newtons).

Statistical results	Experimental Group			

	Control	Ischemia	Ischemia/HBOT	HBOT
p (Shapiro-Wilk)	0.118	0.010	0.107	0.000
Median	0.03	0.03	0.13	0.02
Interquartile range	0.00-0.06	0.01-0.15	0.07-0.27	0.01-0.023
Mean	0.04	0.09	0.17	0.24
Standard deviation	0.04	0.11	0.14	0.54
Standard error	0.01	0.04	0.05	0.17
95% confidence interval				
Lower limit	0.01	0.01	0.05	-0.14
Upper limit	0.07	0.17	0.28	0.62
5% trimmed mean	0.04	0.08	0.16	0.17
Variance	0.00	0.01	0.02	0.29
Minimum	0.00	0.00	0.03	0.00
Maximum	0.13	0.35	0.43	1.72
Range	0.13	0.35	0.40	1.72

HBOT = Hyperbaric Oxygen Therapy

### Histopathology

Colon segments for histopathology were fixed in 10% buffered formalin for 24 hours and embedded in paraffin. Cross-sections of prepared tissue (3 mm thick) were stained with hematoxylin and eosin and evaluated under light microscopy by an experienced pathologist who was unaware of group allocation.

The parameters collagen, fibroblasts, mononuclear infiltrate, polymorphonuclear infiltrate, hemorrhage/congestion, neovascularization, and edema were scored semiquantitatively as absent (0), mild (1), moderate (2), or marked (3) ( Table 2 ). Reepithelialization was scored as absent (0), partial (1), or complete (2) ( Table 3 ). Ulceration, ischemic necrosis, microabscess, bacterial colonies, foreign body, fibrin-leukocyte crust, and separation of wound edges were evaluated dichotomously as absent or present, and were treated as categorical variables ( Table 3 ).

**Table 2:** Relative frequency of histopathology findings stratified by group. Frequencies that were zero in all groups were not listed in the table.

	Experimental Group			

	Control	Ischemia	Ischemia/HBOT	HBOT
Collagen				
Absent	11.11%	14.29%	37.50%	0.00%
Mild	77.78%	85.71%	62.50%	88.89%
Moderate	11.11%	0.00%	0.00%	11.11%
Fibroblast				
Absent	11.11%	0.00%	12.50%	0.00%
Mild	11.11%	14.29%	25.00%	0.00%
Moderate	77.78%	85.71%	62.50%	100.00%
Mononuclear leukocyte infiltrate				
Mild	22.22%	28.57%	12.50%	22.22%
Moderate	77.78%	71.43%	87.50%	77.78%
Polynuclear leukocyte infiltrate				
Absent	11.11%	0.00%	0.00%	0.00%
Mild	11.11%	14.29%	0.00%	11.11%
Moderate	33.33%	14.29%	37.50%	11.11%
Intense	44.44%	71.43%	62.50%	77.78%
Hemorrhage and congestion				
Absent	44.44%	28.57%	50.00%	44.44%
Mild	33.33%	71.43%	50.00%	55.56%
Moderate	22.22%	0.00%	0.00%	0.00%
Neovascularization				
Mild	0.00%	0.00%	37.50%	11.11%
Moderate	100.00%	100.00%	62.50%	88.89%
Edema				
Mild	66.67%	85.71%	75.00%	88.89%
Moderate	33.33%	14.29%	12.50%	11.11%
Intense	0.00%	0.00%	12.50%	0.00%

HBOT = Hyperbaric Oxygen Therapy

**Table 3:** Relative histopathology findings listed by experimental group.

Groups	Control	Ischemia	Ischemia/HBOT	HBOT
Ulceration				
Absent	22%	0%	0%	11%
Present	78%	100%	100%	89%
Fibrin-leukocyte crust				
Absent	33%	0%		11%
Present	67%	100%	100%	89%
Ischemic necrosis				
Absent	89%	100%	75%	78%
Present	11%	0%	25%	22%
Microabscess				
Absent	33%	29%	12%	11%
Present	67%	71%	88%	89%
Bacterial colonies				
Absent	44%	71%	38%	56%
Present	56%	29%	62%	44%
Foreign body				
Absent	89%	100%	75%	100%
Present	11%	0%	25%	0%
Separation of wound edges				
Absent	56%	43%	38%	56%
Present	44%	57%	62%	44%
Reepithelialization				
Absent	44%	43%	75%	33%
Partial	34%	57%	25%	67%
Overall	22%	0%	0%	0%

HBOT = Hyperbaric Oxygen Therapy

### Clinical course

Periorbital contraction, nose and cheek flattening, changes in ear and whisker position, reduced activity, sucked-in flanks, arching of the back, piloerection, and abdominal swelling were evaluated^18 , 19^ . These criteria were evaluated semiquantitatively as imperceptible (0), perceptible (1), or marked (2).

Soon after observation and recording of clinical parameters, rats were weighed on an electronic scale. Clinical observation and weighing were always done at the same time of the day (evening), by the same evaluator, who was blinded to group allocation. Two criteria were established for humane endpoints: weight loss and overall pain score. Individual weight loss greater than 20% of baseline weight at any time of the experiment was defined as a humane endpoint^20^ .

The overall pain score was the sum of the semiquantitative scores for all of the aforementioned clinical criteria during a single evaluation. A pain score of 6 or greater was considered a humane endpoint.

### Statistical analysis

With an alpha = 0.05 and power = 0.85, able to detect a 20% statistical significant difference calculated by T-Test for two samples, the sample size estimated with this effect size was approximately N = 10 for each group. This size group was used in similar studies^4 , 7 , 16^ .

The Shapiro–Wilk test was used to test for normality of distribution. Kruskal–Wallis test was used for compare variables with non-normal distribution. A two-tailed Fisher’s exact test was used to compare the frequency of categorical variables between two groups. To compare change in weight between the first and seventh days of the experiment, the Friedman test for paired nonparametric samples was used. Results are expressed as mean (standard deviation) for samples with normal distribution and as median (first quartile-third quartile) for non-parametric samples^21 - 23^ .

The correlation between all histopathological parameters, without distinction between experimental groups, was calculated pairwise by Kendall’s tau B correlation coefficient (τb) with respective two-tailed p-values.

Statistical significance was considered when p<0.05. The data were analyzed in Microsoft Excel and SPSS for Windows.

## Results

### Survival

One animal in the Ischemia group died during the first operation, and one in the Control group died on the day before reoperation. The reoperation procedures described in the Methods section were performed and samples were collected from the dead animals. At necropsy, no abnormalities were found, and there were no signs of anastomotic leak or peritonitis.

### Body mass

All animals lost weight between the first and seventh days of the experiment (p = 0.02, paired Friedman test). There was no statistically significant difference in relative weight loss across groups (p = 0.921, Kruskal-Wallis test), either on the first day of the experiment (p = 0.619, Kruskal-Wallis test) or on the seventh day (p = 0.990, Kruskal-Wallis test) ( Table 4 ).

**Table 4:** Statistical results of body mass on the first and seventh day of experiment.

	1^st^ of experiment				7^th^ day of experiment			

	Control	Ischemia	Ischemia/HBOT	HBOT	Control	Ischemia	Ischemia/HBOT	HBOT
p (Saphiro wilk)	0.507	0.173	0.046	0.116	0.424	0.148	0.035	0.340
Median	219	218.5	220	222	206	198.5	202	208
First quartile	209	212.50	215.75	218.50	193.25	193.50	196.50	193.25
Third Quartile	237.25	243.75	226.25	230	216	222	214	213

Mean (Standard deviation) weight loss over the course of the experiment was -8.22% (2.73) in the Control group, -8.90% (2.83) in the Ischemia group, -7.69% (2.22) in the HBOT/Ischemia group, and -8.53% (3.19) in the HBOT group. The greatest weight loss recorded was -13.82%. No animal reached the humane endpoint of >20% weight loss.

### Clinical course

No animal reached the humane endpoint criteria established for clinical parameters. The only animal that died in the postoperative period showed only one monitored sign of distress: chromodacryorrhea, classified as 1 (perceptible).

Periorbital contraction, nose and cheek flattening, change in whiskers, arched back, and swollen abdomen were not demonstrated by any animal on any of the days of the experiment. Conversely, chromodacryorrhea was observed in all groups. In one animal from the HBOT/Ischemia group, chromodacryorrhea was scored as 2 (evident); in all others, it was evaluated as 1 (perceptible).

There was no statistically significant difference between groups (p = 0.159 on the fourth day of the experiment, p = 0.778 on the fifth day, p = 0.954 on the sixth day; Kruskal–Wallis test) ( Table 4 ).

Reduced activity was more evident on the fourth day, slight on the fifth day, and not observed on the sixth day. There was no statistically significant difference between groups (p = 0.441 on the fourth day, p = 0.828 on the fifth day, p = 1 on the sixth day; Kruskal–Wallis test) ( Table 5 ).

**Table 5:** Statistical results of clinical parameters on days 4th, 5th and 6th.

Clinical parameter	Experimental Group	4^th^ day		5^th^ day		6^th^ day	

		Mean	Standard deviation	Mean	Standard deviation	Mean	Standard deviation
Chromodacryorrhea	Control	0.78	0.44	0.67	0.50	0.50	0.54
	Ischemia	0.88	0.35	0.88	0.35	0.38	0.52
	Ischemia/HBOT	1.10	0.32	0.70	0.48	0.50	0.53
	HBOT	1.00	0.00	0.70	0.48	0.40	0.52
changes in ear and whisker position	Control	0.00	0.00				
	Ischemia	0.00	0.00				
	Ischemia/HBOT	0.20	0.42				
	HBOT	0.00	0.00				
Reduced activity	Control	0.33	0.50	0.11	0.33		
	Ischemia	0.38	0.52	0.13	0.35		
	Ischemia/OT	0.50	0.53	0.20	0.42		
	HBOT	0.60	0.52	0.20	0.42		
sucked-in flanks	Control	0.22	0.44			0.00	0.00
	Ischemia	0.13	0.35			0.00	0.00
	Ischemia/HBOT	0.20	0.42			0.20	0.42
	HBOT	0.30	0.48			0.10	0.32
Piloerection	Control	0.00	0.00			0.00	0.00
	Ischemia	0.13	0.35			0.00	0.00
	Ischemia/HBOT	0.10	0.32			0.20	0.42
	HBOT	0.10	0.32			0.00	0.00

At least one animal in each group had sucked-in flanks, all classified as 1 (perceptible). There was no statistically significant difference between the groups (p = 0.749 on the fourth day, p = 1 on the fifth day, p = 0.276 on the sixth day; Kruskal-Wallis test) ( Table 5 ).

### Necropsy

On the day of euthanasia, the abdominal cavity was explored for changes such as peritonitis, leakage, and abscesses. Adhesions were evaluated by the Nair score ( Table 6 ). All animals had at least one adhesion. There was no statistically significant difference between groups in this respect (p = 0.202, Kruskal–Wallis test).

**Table 6:** Relative and absolute frequency of animals listed by experimental group, Nair score, and anastomotic leak.

Nair Score	0	1	2	3	4	-	Not evaluated

	Absent	One band	Two bands	Three or more bands	Adhesions to abdominal wall	Anastomotic leak	
Experimental Group							
Ischemia/HBOT	0	0	7 (70%)	3 (30%)	0	0	0
HBOT	0	0	5 (50%)	5 (50%)	0	0	0
Ischemia	0	0	2 (25%)	6 (75%)	0	2	2
Control	0	1 (11.11%)	1 (11.11%)	7 (77.77%)	0	0	1

HBOT = Hyperbaric Oxygen Therapy

Evaluation for anastomotic fistula was performed at the time of necropsy by macroscopic observation of the area surrounding the anastomosis. Fistula was defined as the presence of an orifice with or without leakage of bowel contents. Only animals in the Ischemia group developed fistulas, although there was no statistically significant difference between groups (p = 0.183, Fisher’s exact test).

### Breaking strength

Anastomosis breaking strength presented as median (first quartile- third quartile) was 0.03 (0.00-0.006) N in the Control group, 0.03 (0.01-0.015) N in the Ischemia group, 0.13 (0.07-0.027) N in the HBOT/Ischemia group, and 0.02 (0.01-0.023) N in the HBOT group. There was no statistically significant difference between groups (p = 0.115; Kruskal–Wallis test, three degrees of freedom) ( Table 1 ).

### Histopathology

Means were compared by the Kruskal–Wallis test for independent samples by multiple paired comparisons. There was no statistically significant difference between the groups.

A Kendall’s tau-b correlation analysis was run to determine the relationship between all histopathological parameters without distinction between experimental groups.

Correlation analysis between the parameters showed a strong positive correlation between fibroblasts and collagen (τb = 0.769, p = 0.000) and between microabscesses and polymorphonuclear infiltrate (τb = 0.731, p = 0.000). Correlations were weakly positive between neovascularization and collagen (τb = 0.543, p = 0.002); neovascularization and fibroblasts (τb = 0.534, p = 0.002); reepithelization and collagen (τb = 0.498, p = 0.003); reepithelization and fibroblasts (τb = 0.450, p = 0.008); microabscess and ulceration (τb = 0.352, p = 0.03); fibrin-leukocyte crust and ulceration (τb = 0.528, p = 0.003); fibrin-leukocyte crust and separation of surgical wound edges (τb = 0.383, p = 0.030); and separation of surgical wound edges and fibrin-leukocyte crust (τb = 0.383, p = 0.030). Correlations were weakly negative between fibrin-leukocyte crust and reepithelialization (τb = -0.481, p = 0.005); b) fibrin-leukocyte crust and ulceration (τb = -0.528, p = 0.003); and separation of surgical wound edges and fibroblasts (τb = 0.446, p = 0.010).

Histopathological findings of ulceration, ischemic necrosis, microabscess, microbial colonies, foreign body reaction, and fibrin-leukocyte crust were classified as absent or present. Pairwise between-group comparisons were performed by Fisher’s exact test.

All animals in the Ischemia and HBOT/Ischemia groups showed ulceration and fibrin-leukocyte crust. Although Control animals showed a relatively lower frequency of fibrin-leukocyte crust than HBOT animals (67% *vs* . 89%), there was no statistically significant difference between the groups (p = 0.576, Fisher’s exact test). Control, Ischemia, HBOT/Ischemia, and HBOT animals showed very similar results, confirmed by the absence of a statistically significant difference between the Ischemia and HBOT/Ischemia groups (p = 0.467); or between the Control and HBOT groups (p = 1, Fisher’s exact test).

Microabscesses were found in all groups, with no significant difference (Ischemia *vs* . HBOT/Ischemia, p = 0.569; Control *vs* . HBOT, p = 0.576, Fisher’s exact test).

Bacterial colonies were observed in all groups, again with no statistically significant differences on pairwise comparison (Ischemia vs HBOT/Ischemia, p = 0.315; Control *vs* . HBOT, p = 1, Fisher’s exact test).

No foreign body reaction was found in the Ischemia or HBOT groups. The relative frequency was 11% in the Control group and 25% in the HBOT/Ischemia group; however, there was no statistically significant difference on pairwise comparison (Ischemia vs HBOT/Ischemia, p = 0.467; Control *vs* . HBOT, p = 1, Fisher’s exact test).

Complete reepithelialization occurred in only 22.22% of the control animals, and in no animals in any other groups. There was no statistically significant difference between the Ischemia and HBOT/Ischemia groups in terms of reepithelialization (p = 0.315, Fisher’s exact test).

## Discussion

The hyperbaric protocol of pre and pos intervention sessions at 2.4 ATA failed to produce statistically significant differences, especially in the clinical endpoint of anastomosis leak.

We compared four experimental groups to ascertain the possible benefits of a hyperbaric oxygen therapy protocol consisting of two preoperative sessions and three postoperative sessions on wound healing after end-to-end colonic anastomosis. All animals were thoroughly assessed for a series of clinical parameters, weight loss, macroscopic appearance of the abdominal cavity and anastomosis during reoperation, and histopathological parameters of wound healing, and the breaking strength of the anastomosis was measured directly by a mechanical resistance test.

The use of the surgical microscope for colonic anastomosis was uncomplicated and facilitated visualization, resulting in a more accurate surgical technique. Serial clinical examination revealed only slight changes in all groups, and no animal reached the humane endpoints established for the experiment. Periorbital contraction, nose and cheek flattening, whisker changes, arched back, and swollen abdomen were not demonstrated by any animal on any of the days of the experiment.

The difference in leak frequency between the Ischemia and HBOT/Ischemia groups was not statistically significant. Anastomotic leak from the colonic lumen into the peritoneal cavity developed in only two animals, both in the Ischemia group; no animal in the HBOT/Ischemia, HBOT, or Control groups developed leak. The two animals in the Ischemia group which developed leaks showed no clinical signs of distress other than chromodacryorrhea. One of them presented the highest relative weight loss among all groups (-13.82%), but the second had a weight loss of only -5.8%; this was well short of the mean (standard deviation) for the Ischemia group, which was -8.9 (2.83)%.

Overall mortality in the present study was 5% (n=2/40). One death occurred during anesthesia, while the other occurred postoperatively, on the sixth day of the experiment. The cause of death could not be determined; at necropsy, there was no evidence of bowel obstruction, peritonitis, fistula, or any other abnormality. The survival rate was not statistically significant between groups.

In this study, there was no statistically significant difference in Nair score between groups^24^ and there was no statistically significant difference between groups regarding anastomosis breaking strength.

Histopathologic results were limited to the analysis to one researcher instead of two or more because of human resources constraints of the supporting laboratory. If we had the opportunity to confront data from two or more evaluators we could have estimated inter-rate reliability.

Neither ischemia nor oxygen therapy were associated with alterations that led to a distinct histopathological profile between groups. Induced ischemia did not produce adverse effects that could be evaluated histopathologically on the fourth day. This suggests that the chosen ischemia model was not adequate to produce detectable differences on histopathological parameters this early in the experiment, although two animals in the induced ischemia group developed anastomotic leak.

Groups were statistically identical in terms of mononuclear infiltrate, neovascularization, and edema (p = 1, Kruskal-Wallis). Presence of collagen, fibroblast, and hemorrhage/congestion parameters were also statistically similar in all groups (p = 0.845, p = 0.845, and p = 0.838 respectively, Kruskal–Wallis test) ( Table 2 ).

Previous studies had different results with statistically significant increase in HBOT groups in neovascularization^16 , 27 , 30^ , and in collagen deposition^8^ .

Other two studies corroborate our findings of no significant differences in ischemic necrosis and reepithelization^8 , 16^ .

Analysis of correlation between the parameters of interest revealed a strong positive correlation between fibroblasts and collagen and between microabscesses and polymorphonuclear cell infiltration. This correlation was expected, since fibroblasts synthesize collagen and polymorphonuclear leukocytes have phagocytic effects and induce subsequent production of reactive oxygen and nitrogen intermediates.

There were weak positive correlations between neovascularization and fibroblasts and between neovascularization, and collagen and between fibroblasts and reepithelialization.

Regarding factors that may disrupt healing, there was a weak positive correlation between fibrin-leukocyte crust and ulceration, between separation of wound edges and fibrin-leukocyte crust, and between microabscess and ulceration.

Separation of surgical wound edges correlated negatively with fibroblasts and collagen. Reepithelialization correlated negatively with fibrin-leukocyte crust.

Hyperbaric oxygen therapy has been used as an adjunct in the healing of complicated wounds, pressure ulcers, and diabetic foot lesions. Its use as preconditioning for operations with risk of ischemia and/or ischemia-reperfusion injury has been suggested by some authors^5 , 9 , 10 - 12 , 25^ .

In 2018, a systematic review was published regarding HBOT as an adjunctive therapy for colorectal surgery with the creation of an anastomosis and published between the years 1900 to 2017. Thirteen small animal trials were included, ten studies adopted only postoperative HBOT, one employed combined pre and postoperative, one study compared pre and combined HBOT and just one compared pre with postoperative and with combined HBOT^3^ .

However, there is no consensus on the optimal number of sessions, interval between sessions, interval between the last session and the intervention, and interval between the intervention and the first postoperative session.

There is some evidence that more intensive protocols, defined as more than one session per day, would yield better results^4 , 16 , 26 - 28^ . Maximum pressurization of at least 2.8 ATA^25^ or more days of treatment on previous sessions^30^ has also been shown to yield better results than more conservative protocols with fewer sessions, longer interval between sessions, or lower final pressurization^8 , 29^ .

We can speculate why this experiment did not produce favorable results for the HBOT groups. We believe that sessions with higher maximum pressurization of at least 2.8 ATA could have produced different outcomes, as well as two sessions per day and a decreased lag time before surgery as short as two hours.

We do not infer that the number of preconditioning sessions is one of the causes of the inability to produce more favorable results because other papers have produced results with as few as just one previous session^3^ . Furthermore, maybe more previous sessions could produce some benefit, but a long protocol previous to intervention would not be feasible in clinical situations.

## Conclusion

Based on analysis of anastomotic breaking strength and histopathological features, the hyperbaric oxygen therapy protocol at 2.4 ATA two daily sessions prior to operation as preconditioning and four sessions after it did not influence colonic anastomosis healing on the fourth day after operation.

## Footnotes

1 Research performed at DVM Laboratory Animal Research Unit, Hospital das Forças Armadas (HFA), Brasilia-DF, Brazil. Part of master degree thesis, Postgraduate Program in Medical Sciences, Universidade de Brasília (UnB). Tutor: João Batista de Sousa.
